# Case Report: Behavioral analysis guided intervention targeting triggers and urges in skin-picking disorder with comorbid onychophagia

**DOI:** 10.3389/fpsyt.2025.1738977

**Published:** 2026-01-13

**Authors:** Makoto Kawahito, Keitaro Murayama, Hirofumi Tomiyama, Kenta Kato, Tomohiro Nakao

**Affiliations:** 1 Department of Neuropsychiatry, Graduate School of Medical Sciences, Kyushu University, Fukuoka, Japan; 2 Department of Neuropsychiatry, Kyushu University Hospital, Fukuoka, Japan

**Keywords:** behavioral analysis, case report, habit reversal training, onychophagia, skin-picking disorder, stimulus control

## Abstract

**Background:**

Skin-picking disorder (SPD) often co-occurs with onychophagia and can cause substantial functional impairment. Although evidence-based psychotherapies are effective, benefits may be limited when behavioral analysis is not applied explicitly.

**Case presentation:**

A 29-year-old man with SPD and onychophagia reported marked occupational impact. Baseline self-monitoring showed 240 min/day of nail-related behavior, 30 episodes/day, and high urge intensity. Using behavioral chain analysis, we mapped perceptual antecedents (visual/tactile irregularities) and diurnal variability of urges. A medication-free, nine-session outpatient program over 20 weeks was delivered, combining stimulus control, urge management routines, and habit reversal training. Outcomes were tracked by daily self-monitoring. Rapid improvement followed initiation of stimulus control and consolidated after urge management routines. By treatment end, daily picking time decreased to 15 min/day (-94%), episode frequency to 5/day (-83%), and urge intensity to 4/10 (-60%); no adverse effects were reported.

**Conclusion:**

Making perceptual antecedents and diurnal moderators explicit based on chain analysis enabled a targeted, medication-free intervention that produced clinically meaningful reductions in behavior and urges. A chain-guided behavioral framework may help personalize treatment for SPD and related body-focused repetitive behaviors.

## 1 Introduction

Skin-picking disorder (SPD), also known as excoriation disorder, is characterized by recurrent skin picking that results in significant distress and functional impairment across social, occupational, and financial domains (1, 2). Within the obsessive-compulsive and related disorders (OCRD) spectrum, SPD frequently co-occurs with onychophagia (1, 3). Estimates of SPD prevalence range from 1.4% to 5.4%, with onset typically occurring during adolescence (4). Although patients with SPD have a lower quality of life than healthy controls, less than one-fifth seek treatment (2, 3). Psychotherapy is an evidence-based intervention for treating SPD. Psychotherapeutic approaches include cognitive-behavioral therapy (CBT), habit reversal training (HRT), and acceptance and commitment therapy (ACT) (5). CBT generally involves psychoeducation, cognitive restructuring, and an emphasis on relapse prevention (4, 6). HRT includes awareness and competing response training (7). ACT promotes the acceptance of negative thoughts and feelings, and focuses on personal values and goals (8). Although these approaches are effective, their benefits may be constrained when sensory antecedents and internal states are not explicitly assessed or targeted (9).

We report a medication-free, brief outpatient intervention for SPD that systematically maps perceptual antecedents (visual/tactile irregularities) and diurnal variability in urges using behavioral chain analysis and then applies focused stimulus control and urge management procedures. This case illustrates rapid clinical improvement over nine sessions and highlights how making antecedents explicit can enhance behavioral interventions for SPD and related body-focused repetitive behaviors (10, 11). Key outcomes were tracked with structured self-monitoring, and the methodological details and measures are described below.

## 2 Case description

### 2.1 Patient information

A 29-year-old right-handed man presented with chronic skin-picking and nail-biting since childhood, which caused substantial occupational impairment. The patient worked long hours in a high-demand professional setting and reported spending prolonged periods each day engaging in nail-related behaviors at work. The patient attempted to conceal the behavior but was unable to stop it. There was no history of neurodevelopmental, psychiatric, mood, or substance use disorders, or other relevant medical illnesses. The patient had no family history of OCRDs.

### 2.2 Clinical findings

**Figure 1:**
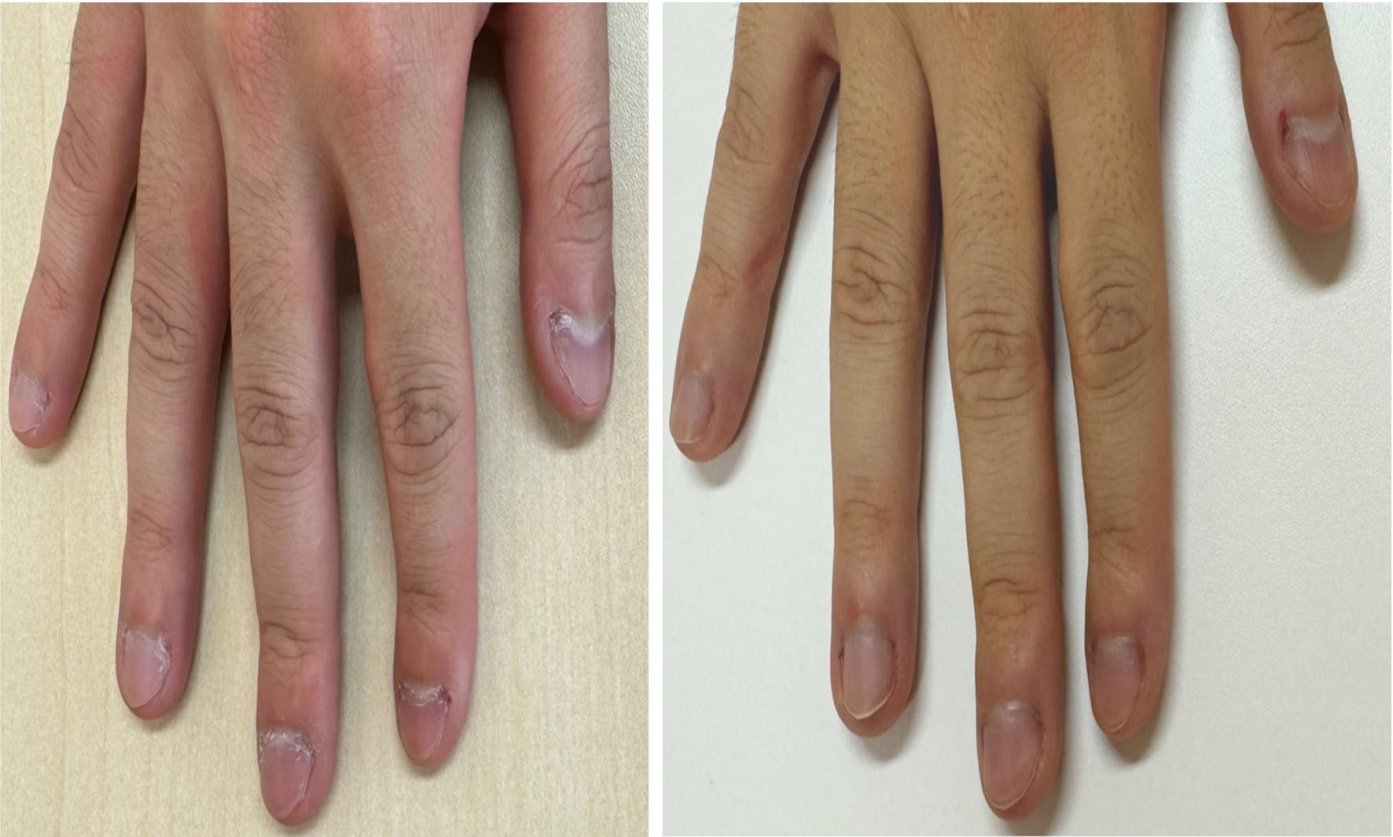
Baseline and post-treatment nail appearance. Left: At baseline (Session 1), free edges of nail plates are not visible; marked peeling of lateral nail folds. Right: After Session 9, free edges are visible with reduced periungual peeling.

This behavior involved only the fingernails and adjacent periungual skin. The nails were shortened so that the free edges of the nail plates were no longer visible, with marked peeling of the lateral nail folds (**Figure 1**). No distinct dermatological conditions requiring specific treatment were identified. The patient expressed shame and distress regarding loss of control and work delays. At baseline, self-monitoring indicated approximately 240 min/day of nail-related behavior, with a frequency of approximately 30 episodes per day. The self-rated urge intensity (0–10 scale) was 10 at baseline. These measures were the median values between visits. Episodes typically begin with the tactile and visual detection of uneven skin/nails, followed by an escalating urge, picking/biting, and transient relief.

## 3 Diagnostic assessment

The patient met the Diagnostic and Statistical Manual of Mental Disorders, Fifth Edition (DSM-5) criteria for SPD, which is characterized by recurrent skin picking with unsuccessful attempts to decrease or stop, clinically significant distress, and functional impairment (1). Onychophagia, which is not classified as a separate disorder in the DSM-5, also presents as nail-focused repetitive behavior (12). At the diagnostic assessment, no other mental disorders, including OCRDs, anxiety disorders, or depressive disorders, were identified in the clinical interviews.

We used daily self-monitoring with three indices (duration, episode frequency, and urge intensity) to quantify the behavioral output and subjective urges. Symptom severity and impact in SPD are commonly assessed with the Skin Picking Scale (SPS) and the Skin Picking Impact Scale (SPIS) (13–15). SPS comprises six items assessing: (i) frequency of urge to pick, (ii) intensity of urge to pick, (iii) time spent picking, (iv) interference due to skin picking, (v) distress due to picking/distress when prevented from picking, and (vi) avoidance behavior due to picking (13). The SPIS includes ten items capturing psychosocial consequences, dissatisfaction with appearance, and negative self-evaluation (14, 15). However, because validated Japanese versions of the standardized SPD scales were not available in our setting at the time of assessment, we selected these three SPS-aligned indices as our primary outcomes. This pragmatic approach allowed us to track changes in behavioral output and subjective urges.

At the outset, the diagnosis and evidence-based treatment options were explained, and the treatment goals were aligned. The patient subsequently engaged in nine outpatient behavioral sessions for 20 weeks (approximately 30 min each, scheduled every 2–4 weeks).

## 4 Therapeutic intervention and outcomes

The first author (M.K.), a psychiatrist, conducted nine brief outpatient behavioral sessions that were conducted over 20 weeks without pharmacotherapy (**Table 1**). The intervention sequence was individualized based on functional chain analysis and iteratively refined based on patient self-monitoring of min/day, episodes/day, and urge intensity (0–10).

**Table 1:** Timeline of the nine-session intervention and outcomes across 20 weeks.

Sessions	Key components	Details	Picking time (min/day)	Episode frequency (episodes/day)	Urge intensity (0-10)
1	Psychoeducation & self-monitoring	Explain disorder, align goals. Initiate self-monitoring after each episode.	240	30	10
2	Behavioral analysis & HRT	Antecedents not yet identified. Introduce competing responses (gum/gummy candy; hand-sized ball).	210	28	9
3	Behavioral analysis & Stimulus control (initiated)	Logs clarified that episodes began with tactile and visual detection of uneven skin/nail, followed by escalating urge, then picking/biting and transient relief. Begin smoothing/covering (hand cream; adhesive bandages).	200	26	9
4	Stimulus control (continued)	Marked reduction in daily duration and frequency.	90	13	7
5	Urge-management (initiated)	Addressed diurnal variability: added a 15-min noon nap; scheduled late-day music/talk radio. Continued stimulus-control at work.	60	10	6
6	Urge-management (continued) & chain refinement	Gains consolidated.	30	10	6
7	Lifestyle management	Temporary increase with peak workload/irregular sleep; implemented a fixed sleep schedule guided by the mapped chain.	60	12	6
8	Relapse prevention	Reviewed self-monitoring logs and the one-page chain diagram; reinforced relapse-prevention routines.	30	10	5
9	Maintenance	Review logs/chain diagram; relapse-prevention plan.	15	5	4

Nine brief outpatient behavioral sessions were conducted over 20 weeks (approximately 30 min each, every 2–4 weeks). Values are medians of daily self-monitoring recorded between consecutive visits: picking time (min/day), episode frequency (episodes/day), and urge intensity (0-10). Baseline = Session 1. HRT, habit reversal training.

Session 1–2. M.K. provided psychoeducation that frames skin-picking and nail-biting as treatable behaviors, normalizes help-seeking, and aligns functional goals (improved control and reduced time loss at work). While the patient initially attributed his symptoms to stress or personality deficits, he reported significant relief upon re-conceptualizing the behavior as a treatable disorder serving a sensory regulatory function. In Session 1, a structured daily self-monitoring log was introduced. The patient was instructed to record entries immediately following each picking/biting episode. The log components included: (i) time of day, (ii) duration of the behavior, (iii) urge intensity (rated 0–10), and (iv) situational context/triggers. Habit reversal elements, including competing responses (chewing gum or gummy candy, and squeezing a hand-sized ball), were introduced. However, the antecedents had not yet been identified. Psychoeducation reduced distress, self-monitoring clarified behavioral patterns, and competing responses delayed initiation; however, the overall daily duration and frequency showed little change during this phase (**Table 1**).

**Figure 2:**
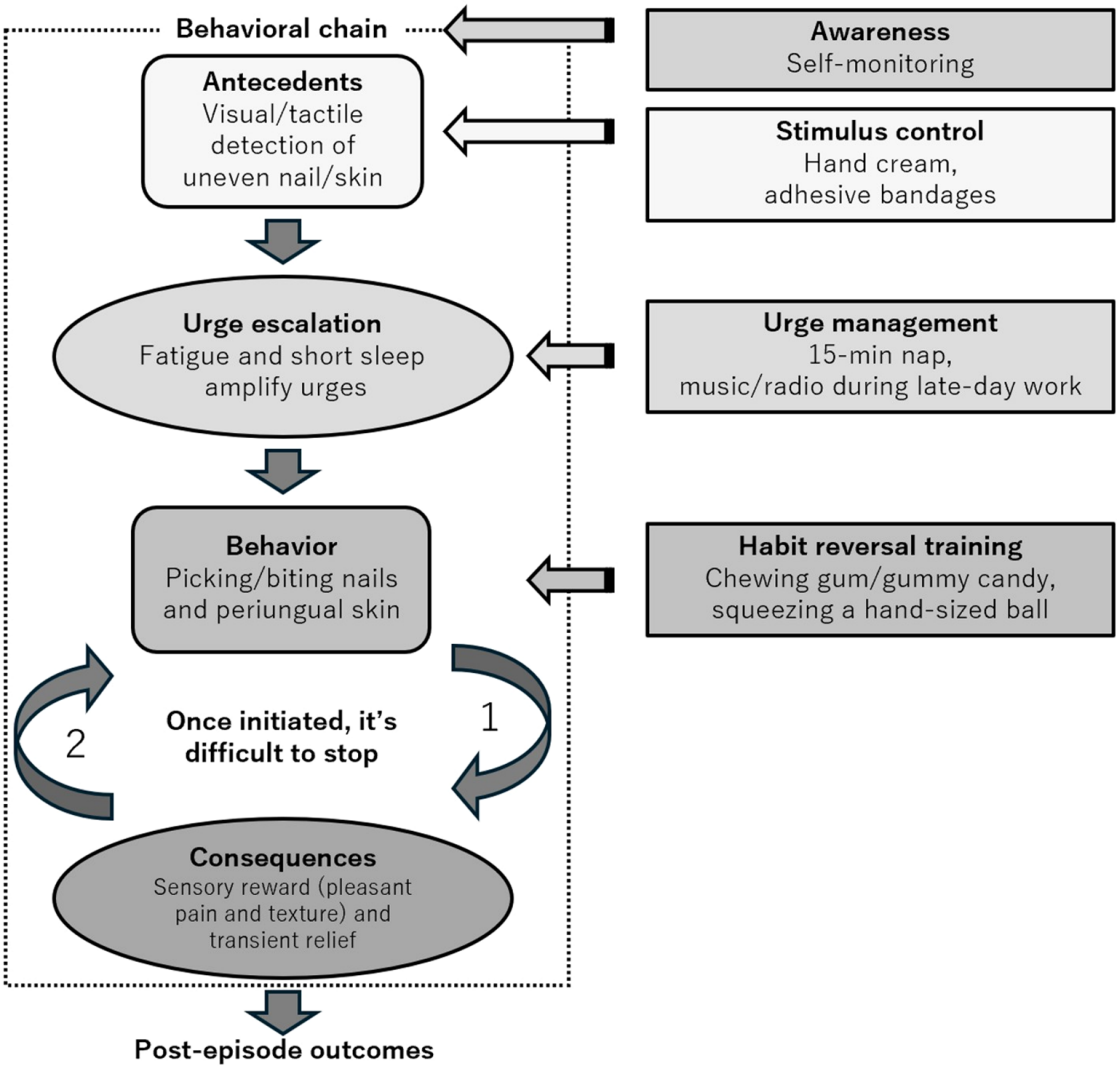
Behavioral analysis and targeted interventions. Self-monitoring reveals a chain beginning with perceptual antecedents (visual/tactile irregularities), followed by urge escalation (amplified by fatigue and short sleep), resulting in picking/biting of nails and periungual skin, transient sensory relief, and shame/distress post-episode. Interventions are mapped at each step: awareness/self-monitoring, stimulus control (hand cream, adhesive bandages), urge management (15-minute noon nap; music/radio late in the day), and HRT (habit reversal training) competing responses (chewing gum/gummy candy; squeezing a hand-sized ball).

**Figure 3:**
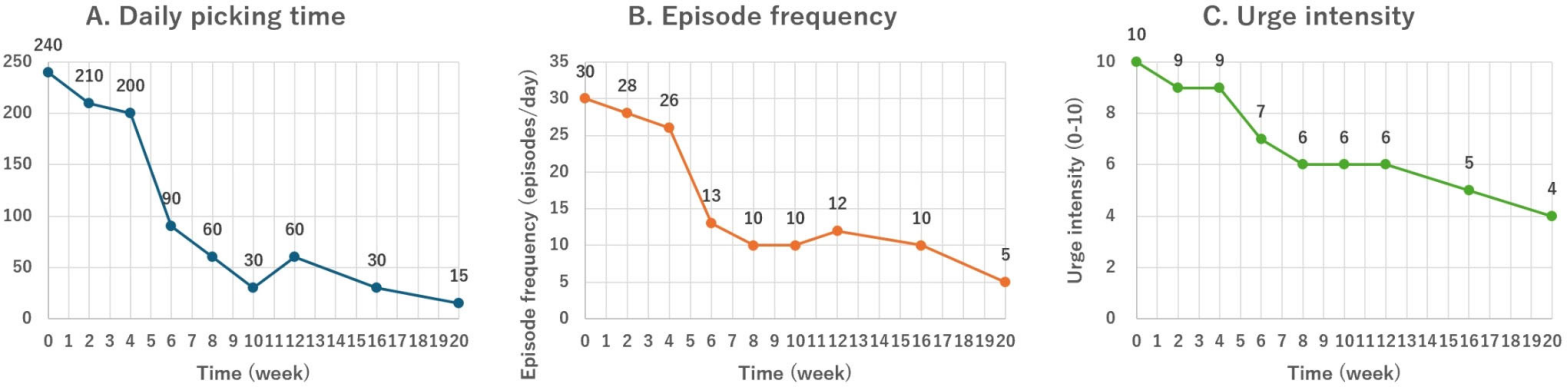
Daily self-monitoring outcomes across the 20-week intervention. **(A)** Daily picking time (min/day), **(B)** episode frequency (episodes/day), and **(C)** urge intensity (0–10). Points represent medians of daily entries between consecutive visits; week 0 = baseline (Session 1), week 20 = post-treatment (Session 9).

Session 3–4. Repeated logs revealed a consistent behavioral chain: tactile/visual detection of periungual irregularities led to an escalating urge, followed by picking/biting and transient relief (**Figure 2**). Based on this formulation, stimulus control targeting perceptual antecedents was implemented, consisting of smoothing/covering strategies (regular use of hand cream and adhesive bandages, as needed) to reduce the salience of uneven nail/skin surfaces. A rapid response ensued: daily picking time declined to 90 min/day by Session 4, and episode frequency was nearly halved (**Figure 3**).

Session 5–6. Because urges showed diurnal variability and worsened with evening fatigue and short sleep, a brief 15-minute planned nap at noon was added, and listening to music/talk radio during late-day work periods was scheduled. Combined with ongoing stimulus control, these procedures consolidated gains with a daily picking time of approximately 30–60 min/day and reduced episode counts (**Figure 3**). During this phase, a behavioral chain diagram (**Figure 2**) was developed and refined.

Session 7. A temporary increase in symptoms coincided with peak workload and irregular sleep, which amplified evening urges. A fixed sleep schedule was instituted, guided by a previously mapped behavioral chain.

Session 8–9. The patient reviewed self-monitoring notes and a one-page behavioral chain diagram. The patient reported improved insights into his behavioral chain and treatment rationale. By the end of the treatment, the daily picking time had decreased from 240 to 15 min/day (-94%), episode frequency from 30 to 5 episodes/day (-83%), and urge intensity from 10 to 4 (-60%). These improvements were maintained throughout the treatment period. No adverse events were observed.

## 5 Patient perspective

“For years, I believed that stopping this habit was simply a matter of willpower, and I felt ashamed every time I failed. Identifying my specific triggers was a turning point. Seeing my behavior chain mapped out helped me understand the ‘why’ and ‘how’ of my picking aimed at sensory regulation, rather than just blaming my personality. Simple prevention steps improved my appearance and daily life. Having a clear logic made it easier to manage urges before they escalated.”

## 6 Discussion

This single-case report illustrates a mechanism-informed behavioral formulation and intervention for skin-picking disorder with comorbid onychophagia. By mapping perceptual antecedents (visual/tactile irregularities) and temporal moderators (fatigue and short sleep) through chain analysis, we selected targeted stimulus control and urge management procedures. A rapid improvement followed the antecedent-focused strategies, and gains were consolidated when sleep and late-day attention shift routines were introduced, without pharmacotherapy.

Automatic and focused patterns of skin-picking are often discussed. The automatic pattern (less conscious and weakly articulated antecedents) tended to respond to the HRT and upstream antecedent interventions. In contrast, the focused pattern is enacted intentionally to modulate internal states (e.g., anxiety, tension, and disgust) and is theoretically aligned with ACT-consistent procedures (16). In clinical practice, many patients exhibit both features. Thus, an early priority is to analyze the behavior, identify the functional chain, and select the most appropriate intervention for each process (**Figure 2**). Previous studies suggested that combined protocols could yield additional improvements (16). In this case, self-monitoring helped make the behavioral chain explicit, stimulus control targeted antecedents, urge management strategies addressed urges, and HRT provided competing responses to replace maladaptive behaviors.

A clinically meaningful asynchrony emerged: the time spent and the frequency of picking/biting decreased substantially, whereas urge intensity decreased to a lesser extent. Conceptually, stimulus control and HRT are well-suited to disrupt cue-response chains and reduce overt behavioral output, but they may not directly modify the subjective urge experience, which is shaped by private events and state-dependent factors. Considering this pattern, adding ACT components might yield further reductions in the urge intensity.

The strengths of this case include: (i) a mechanism-informed, chain-guided formulation that directly determines intervention selection and sequencing, offering a transparent and replicable rationale; (ii) daily monitoring with three complementary indices—duration, episode frequency, and self-rated urge intensity—which detected the clinically relevant asynchrony between reductions in behavioral output and the change in subjective urges; and iii) clinically meaningful gains achieved without medication, using affordable, scalable methods suitable for standard care.

The limitations include those inherent to a single case report without condition control: potential threats to internal validity (history, maturation, measurement reactivity, and expectancy), reliance on self-reports without independent blinded ratings, and a short follow-up, leaving durability and relapse risk uncertain. Because multiple components were introduced sequentially, we could not isolate the active ingredients or determine the optimal order; therefore, an alternative sequence might have produced different results. Generalizability is constrained by the behavior’s localization to the nails and periungual skin and the single-provider setting. Finally, quality of life and functional impairment were not measured using standardized instruments, limiting inferences about broader functioning.

## 7 Conclusion

In patients with SPD and onychophagia, a behavioral chain analysis that explicitly mapped perceptual antecedents and diurnal variability guided a targeted, medication-free intervention. Combining stimulus control with urge management produced a rapid reduction in daily picking time, episode frequency, and urge intensity, which was maintained throughout the treatment period. These findings highlight the value of mechanism-informed, chain-guided behavioral analyses for SPD.

## Data Availability

The raw data supporting the conclusions of this article will be made available by the authors, without undue reservation.
